# Predictors of skilled birth attendance among married women in Cameroon: further analysis of 2018 Cameroon Demographic and Health Survey

**DOI:** 10.1186/s12978-021-01124-9

**Published:** 2021-03-25

**Authors:** Sanni Yaya, Betregiorgis Zegeye, Bright Opoku Ahinkorah, Abdul-Aziz Seidu, Edward Kwabena Ameyaw, Nicholas Kofi Adjei, Gebretsadik Shibre

**Affiliations:** 1 School of International Development and Global Studies, University of Ottawa, Ottawa, Canada; 2 The George Institute for Global Health, Imperial College London, London, UK; 3 HaSET Maternal and Child Health Research Program, Shewarobit Field Office, Shewarobit, Ethiopia; 4 School of Public Health, Faculty of Health, University of Technology Sydney, Sydney, NSW Australia; 5 Department of Population and Health, University of Cape Coast, Cape Coast, Ghana; 6 College of Public Health, Medical and Veterinary Sciences, James Cook University, Townsville, QLD Australia; 7 Leibniz Institute for Prevention Research and Epidemiology, BIPS, Heiligenhafen, Germany; 8 Department of Reproductive, Family and Population Health, School of Public Health, Addis Ababa University, Addis Ababa, Ethiopia

**Keywords:** Predictors, Skilled birth attendance, Cameroon, DHS, Global health

## Abstract

**Background:**

In Cameroon, maternal deaths remain high. The high maternal deaths in the country have been attributed to the low utilization of maternal healthcare services, including skilled birth attendance. This study examined the predictors of skilled birth services utilization among married women in Cameroon.

**Methods:**

Data from the 2018 Cameroon Demographic and Health Survey was analyzed on 7881 married women of reproductive age (15–49 years). Both bivariate and multivariable logistic regression analyses were carried out to determine the predictors of skilled childbirth services. The results were presented with crude odds ratio (cOR) and adjusted odds ratio (aOR) and 95% confidence interval (CI).

**Results:**

The coverage of skilled birth attendance among married women of reproductive age in Cameroon was 66.2%. After adjusting for potential confounders, media exposure (aOR = 1.46, 95% CI: 1.11–1.91), higher decision making (aOR = 1.88, 95% CI: 1.36–2.59), maternal education (aOR = 2.38, 95% CI; 1.65–3.42), place of residence (aOR = 0.50, 95% CI; 0.33–0.74), religion (aOR = 0.55, 95% CI; 0.35–0.87), economic status (aOR = 5.16, 95% CI; 2.58–10.30), wife beating attitude (aOR = 1.32, 95% CI; 1.05–1.65), parity (aOR = 0.62, 95% CI; 0.41–0.93) and skilled antenatal care (aOR = 14.46, 95% CI; 10.01–20.89) were found to be significant predictors of skilled birth attendance.

**Conclusions:**

This study demonstrates that social, economic, regional, and cultural factors can act as barriers to skilled childbirth services utilization in Cameroon. Interventions that target women empowerment, antenatal care awareness and strengthening are needed, especially among the rural poor, to reduce barriers to care seeking. Maternal healthcare services utilization interventions and policies in Cameroon need to focus on specific equity gaps that relate to socio-economic status, maternal education, and the economic empowerment of women. Such policies and interventions should also aim at reducing geographical barriers to access to maternal healthcare services, including skilled birth attendance. Due to the presence of inequities in the use of skilled birth attendance services, programs aimed at social protection and empowerment of economically disadvantaged women are necessary for the achievement of the post-2015 targets and the Sustainable Development Goals.

**Plain English Summary:**

Globally, Cameroon is one of the countries with high maternal deaths. Low utilization of maternal healthcare services, including skilled birth attendance have been found to account for the high maternal deaths in the country. This study sought to examine the factors associated with skilled childbirth services utilization among married women in Cameroon.

Using data from the 2018 Cameroon Demographic and Health Survey, we found that the coverage of skilled birth attendance among married women of reproductive age in Cameroon is high. Factors such as higher decision-making power, higher maternal education, place of residence, religion, higher economic status, wife beating attitude, parity and skilled antenatal care were found to be the significant predictors of skilled birth attendance.

This study has shown that socio-economic, regional and cultural factors account for the utilization of skilled childbirth services utilization in Cameroon. Interventions aimed at enhancing the utilization of skilled childbirth services in Cameroon should target women empowerment, antenatal care awareness creation and sensitization, especially among the rural poor, to reduce barriers to care seeking. Maternal healthcare services utilization interventions and policies in Cameroon need to focus on specific equity gaps that relate to socio-economic status, maternal education, and the economic empowerment of women.

## Background

Approximately 810 women die every day from pregnancy and child birth related causes globally [1], and about 94% of these deaths occur in low and middle-income countries [1]. In 2017, sub-Saharan Africa alone accounted for approximately two-thirds (196 000) of maternal deaths [1]. Over the past years, maternal mortality has declined in Sub-Saharan Africa [1]. Despite the decline, it appears most countries in the sub-region have made little efforts in achieving the Sustainable Development Goal 3.1, which seeks to ensure a reduction in the global maternal mortality ratio to less than 70 per 100 000 live births by 2030 [2]. In Cameroon for instance, the maternal mortality ratio increased from 669 in 2006 to 782 in 2011 [3–6]. A recent report by the WHO, UNICEF and UNFPA indicate a maternal mortality ratio of 529 per 100, 000 live births in 2017 in Cameroon [7].

Reports show that skilled birth attendance can considerably decrease maternal and neonatal mortality [8], and other obstetric complications, including stillbirths [8, 9]. Skilled birth attendants (SBAs) are qualified health professionals (i.e., midwives, doctors, or nurses) who have been provided with education and training to capably attend to normal (uncomplicated) pregnancies, childbirths and the immediate postnatal period [10]. They are also trained to handle the identification, management and referral of complications in women and newborns [10]. Thus, skilled birth attendance is vital and an optimal strategy for preventing maternal and perinatal mortality [11–13]. Globally, about 81 percent of births are attended by skilled professionals [14], while only 60 percent of skilled birth attendance occur in sub-Saharan-Africa [14].

In Cameron, maternal death remains high [3, 4], yet the coverage of skilled childbirth services is only 70% [15]. This could be attributed to low birth preparedness and complication readiness [16], which can be explained by poor antenatal care utilization, low income [16], and shortage of health care providers [17], which is complicated by unequal distribution across regions [17].

Skilled birth attendance is largely influenced by socioeconomic, cultural and other related factors [15, 16, 18–25]. However, there is a paucity of evidence about predictors of skilled birth attendance in Cameron. The aim of this study is to explore the predictors of skilled birth services utilization in Cameroon using data from the recent nationally representative demographic and health survey.

This study is underpinned by Anderson and Newmans’ Health Care Utilisation Model which proposes that the use of a service, which includes skilled birth attendance is influenced by predisposing factors such as demographics, health beliefs and social structures; enabling factors which include the availability of health personnel and facilities and waiting time and financial assistance; and need for care factors which focuse on factors which are people’s perception and evaluation of their health that serve as motivation to use a service [26]. In line with this theoretical framework, our interest was to understand how socio-demographic (maternal age, education, husband’s education an occupation, employment status, parity, wealth index), health beliefs (religion), social structures (exposure to media, region, decision-making capacity, attitude towards wife beating) and access to health services (skilled antenatal care) play a role in the use of skilled childbirth services.

## Methods

### Study area

Located in Central Africa, Cameroon is a lower-middle-income country with a population of over 25 million as of 2018 [27, 28]. It is divided into northern, central, southern, and western geographic regions [27, 28] and the largest economy in the Central African Economic and Monetary Community (CEMAC), a region notable of an economic crisis that emanated from the steep fall in oil prices [28]. Economic growth in Cameroon was estimated to reach 4.3% in 2019 [28], while the country’s real Gross Domestic Product (GDP) was estimated to grow by approximately 8% (or 5.7% per capita) between 2015 and 2035 [28]. In Cameroon, primary health care (PHC) is provided in line with the health district framework proposed by the World Health Organization (WHO) Regional Office for Africa, entailing a nurse-based, doctor-supported infrastructure of State-owned, denominational and private integrated health centers [29]. It is supported by a diverse and fragmented system of community health workers recruited by priority public health vertical programmes [29]. The 2016 evaluation of this sectoral strategy found that 7% of the 189 health districts were serviced [29].

## Data

Data for this study were obtained from the 2018 Cameron Demographic and Health Survey (CDHS). This survey was conducted by the National Institute of Statistics (NIS), in collaboration with the Ministry of Public Health [15], the United States Agency for International Development (USAID), and other national and global institutions [15]. The CDHS was carried out using a two-stage cluster sampling design based on enumeration areas and household samples. The first stage involved the selection of enumeration areas with probability proportional to size. At the second stage, household sampling was done where 13,527 women aged 15–49 years and 6,978 men aged 15–64 years were interviewed respectively [15]. Detailed methodology of the CDHS has been outlined in the final report of the 2018 CDHS [15]. In this study, the analysis was restricted to currently married women with at least one birth in the five years prior to the survey [30]. We included only married women in this study because we were interested in how components of women empowerment such as decision making power and attitude towards wife beating [31], affect a woman’s decision to access skilled assistance during delivery and data on these variables were available for only married women.

## Variables

### Dependent variable

The outcome variable of interest was skilled birth attendance. In this study, a birth was considered as skilled birth attendance if it is attended by a skilled health personnel (doctor, nurse, and midwife and auxiliary midwife) [14] and this was dichotomized and coded based on the assistance of delivery (skilled birth attendant = 1, unskilled attendant = 0).

## Independent variables

The independent variables considered in this study were maternal age [15–49], maternal education (no formal education, primary school, secondary school, higher), religion (Catholic, Protestant, Other Christians, Muslim, Animist, Others), region (Adamawa, Centre (Without Yaounde), Douala, East, Far-North, Littoral (Without Douala), North, North-West, West, South, South-West, Yaounde), place of residence (urban, rural), husband education (no formal education, primary school, secondary school, higher) and parity (1–2, 3–4, ≥ 5). Media exposure (newspaper, radio or television) was either no exposure to any of the three or exposure to at least one of the three at least once a week. Skilled antenatal care (ANC) was coded as “yes” if a woman had an ANC follow up by doctor, nurse/midwife, axillary midwife, and coded as “no” if otherwise.

Other explanatory variables included were wealth index (poorest, poorer, middle, richer, richest). Decision-making and attitudes towards wife beating were also included as a proxy indicator of women empowerment [24]. The DHS asks respondents about their decision-making power regarding several dimensions: making decisions regarding own health care, large household purchases and visits to family or relatives. In this study, if the decision on all three dimensions was made by husband/partner alone or by other (i.e., mother in law), it was coded as no decision making, if a respondent had decision-making power alone or with her husband/partner over one or two of the decision-making parameters, it was coded as moderate decision making and if a respondent had decision-making power either alone or with her husband on three of the decision-making parameters, it was coded as higher decision making. With attitude towards wife beating, women were asked of a husband’s justification of wife beating for the following reasons: (i) burning food (ii) arguing with him (iii) going out without telling him (iv) neglecting the children, and (v) refusing to have sexual intercourse with him. A binary variable was created to reflect attitude towards wife beating. Attitude towards wife beating was coded as ‘no’ if respondent did not agree with wife beating, and ‘yes’ if she accepted wife beating as normal/healthy behaviour.

## Data analysis

The data analysis comprised of both descriptive and multivariable logistic regression analyses. First, we estimated the frequencies and percentages for all the variables included in the analysis. Secondly, bivariate logistic regression was performed and statistically significant (*P*-values; *P* < 0.05) variables were further included in a multivariable logistic regression model to determine the predictors of skilled childbirth services after checking for multicollinearity using variance inflation factor (VIF) (Max = 8.56, Min = 1.09, Mean = 3.02). The final adjusted outputs were reported using crude odds ratio (cOR) and adjusted odds ratio (aOR) at 95% confidence interval (CI). The dataset was weighted to account for differences in sampling design. This procedure mitigates inflated type one errors and large CIs. All statistical analyses were performed in Version 14 (Stata Corp, College Station, Texas, USA). All frequency distributions were weighted (v005/1,000,000) while the survey command (SVY) in Stata was used to adjust for the complex sampling structure of the data in the regression analyses.

### Ethical considerations

This study was based on a secondary dataset with no identified information on the participant. The authors obtained and were granted approval to use the dataset by MEASURE DHS.

## Results

### Socio-demographic characteristics

Table 1 shows the distribution of respondents’ characteristics. A total of 7,881 married women were included in the analyses. Approximately 58% of the respondents were rural residents, and about 30% of the women were in 25–29 years age group. More than two sixth of the respondents attended primary school (34.3%) and secondary school (34.7%). Approximately 34% of the participants were Catholics, Muslims (29.9%) and Protestants (25.3%). Majority (86.1%) of them had skilled ANC visit. Regarding women empowerment, about 32% of the respondents had no decision-making power either alone or together with their husband on all of the three decision making power parameters (i.e., her own health, to purchase large household expense, to visit family or relatives). About 70% of the participants did not accept wife beating on all the five wife beating reasons: burning food, arguing husband, goes out without telling husband, neglecting children, and refuse to sex.

**Table 1:** Respondent characteristics and distribution of skilled birth attendance, Cameron 2018 DHS

Variables	Frequency	Percent	Skilled birth attendance	
			Yes	No
Maternal age				
15–19	542	6.9	206 (38.0)	336 (62.0)
20–24	1660	21.1	522 (31.5)	1138 (68.6)
25–29	2293	29.1	712 (31.1)	1581 (69.0)
30–34	1778	22.6	521 (29.3)	1257 (70.7)
35–39	1105	14.0	335 (30.3)	770 (69.7)
40–44	411	5.2	137 (33.3)	274 (66.7)
45–49	92	1.8	43 (46.7)	49 (53.3)
Maternal education				
No formal education	2075	26.3	1397 (67.3)	678 (32.7)
Primary school	2705	34.3	810 (29.9)	1895 (70.1)
Secondary school	2734	34.7	262 (9.6)	2472 (90.4)
Higher	367	4.7	7 (1.91)	360 (98.1)
Husband education				
No formal education	1883	23.9	1127 (59.6)	756 (40.2)
Primary school	2600	33.0	895 (34.4)	1705 (65.6)
Secondary school	2790	35.4	435 (15.6)	2355 (84.4)
Higher	608	7.7	19 (3.13)	589 (96.9)
Place of residence				
Urban	3332	42.3	392 (11.8)	2940 (88.2)
Rural	4549	57.7	2084 (45.8)	2465 (54.2)
Religion				
Catholic	2637	33.5	553 (21.0)	2084 (79.0)
Protestant	1991	25.3	521 (26.2)	1470 (73.8)
Other Christians	608	7.7	170 (28.0)	438 (72.0)
Muslim	2353	29.9	1062 (45.1)	1291 (54.9)
Animist	130	1.7	98 (75.4)	32 (24.6)
Others	162	2.1	72 (44.44%)	90 (55.56%)
Economic status				
Poorest	1671	21.2	1172 (70.1)	499 (29.9)
Poorer	1849	23.5	774 (41.9)	1075 (58.1)
Middle	1770	22.5	392 (22.2)	1378 (77.9)
Richer	1500	19.0	110 (7.3)	1390 (92.7)
Richest	1091	13.8	28 (2.6)	1063 (97.4)
Media exposure				
No	3562	45.2	1956 (54.9)	1606 (45.1)
Yes	4319	54.8	520 (12.0)	3799 (88.0)
Region				
Adamawa	653	8.3	358 (54.8)	295 (45.2)
Centre (Without Yaoundé)	843	10.7	172 (20.4)	671 (79.6)
Douala	411	5.2	14 (3.4)	397 (96.6)
East	768	9.7	334 (43.5)	434 (56.5)
Far-North	1213	15.4	731 (60.3)	482 (39.7)
Littoral (Without Douala)	416	5.3	18 (4.3)	398 (95.7)
North	1134	14.4	655 (57.8)	479 (42.2)
North-West	366	4.6	29 (7.9)	337 (92.1)
West	796	10.1	22 (2.8)	6774 (97.2)
South	664	8.4	122 (18.4)	542 (81.6)
South-West	119	1.5	1 (0.8)	118 (99.2)
Yaoundé	498	6.3	20 (4.0)	478 (96.0)
Skilled ANC				
No	695	13.9	613 (88.2)	82 (11.8)
Yes	4318	86.1	866 (20.1)	3452 (79.9)
Decision making				
No decision making	2521	32.0	1233 (48.9)	1288 (51.1)
Decision making one	1813	23.0	482 (26.59%)	1331 (73.4)
Decision making two	3547	45.0	761 (21.45%)	2786 (78.6)
Attitude towards wife-beating				
Accept	2407	30.5	866 (36.0)	1541 (64.0)
Refuse	5474	69.5	1610 (29.4)	3864 (70.6)***
Parity				
1–2	2287	29.0	569 (24.9)	1718 (75.1)
3–4	2647	33.6	760 (28.7)	1887 (71.3)
5+	2947	37.4	1147 (38.9)	1800 (61.1)

### Prevalence of skilled birth attendance

**Fig. 1:**
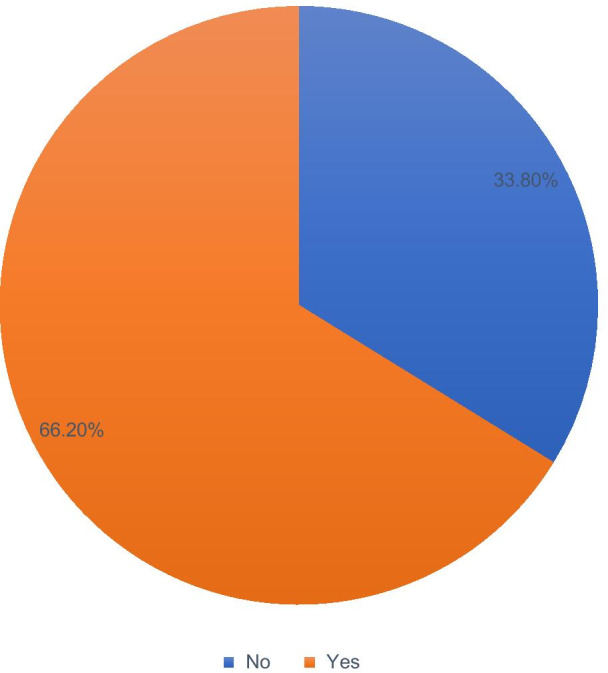
Coverage of skilled delivery service utilization among married women in Cameroon, Demographic and Health Surveys 2018

The coverage of skilled childbirth services utilization among married women was 66.2% (Fig. 1).

### Predictors of skilled childbirth services utilization

The results in Table 2 show the predictors of skilled childbirth services utilization in Cameroon. The results indicate that women who had primary school education reported higher odds of skilled birth attendance (aOR = 1.46, 95% CI; 1.13–1.90), compared to those with no formal education. With place of residence, married women living in rural areas were less likely (aOR = 0.50, 95% CI; 0.33–0.74) to report skilled birth attendance, compared to their counterparts in urban areas. In terms of religion, the utilization of skilled childbirth services was lower for other Christians (aOR = 0.55, 95% CI; 0.35–0.87) religious group, compared to Catholics. Regarding household economic status, we observed that skilled childbirth services utilization was higher among married women in the richest wealth index (aOR = 5.16, 95% CI; 2.58–10.30), compared to the poorest. Utilization of skilled childbirth services was significantly influenced by media exposure. Married women who were exposed to media (i.e., newspapers, radio or television for at least less than once a week) were more likely (aOR = 1.46, 95% CI; 1.11–1.91) to use skilled childbirth services than those with no media exposure. Furthermore, we found subnational differences, where utilization of skilled childbirth services was found to be higher among married women in the Littoral (Without Douala) region (aOR = 10.89, 95% CI; 4.57–25.95) and West region (aOR = 9.86, 95% CI; 4.41–22.05), compared to Adamawa region.

**Table 2:** Predictors of skilled birth attendance among married women In Cameroon, 2018

Variables	cOR	p-values	aOR (95% CI)	p-values
Maternal age				
15–19	Ref		Ref	
20–24	1.24 (0.94–1.63)	0.115	0.82 (0.55–1.23)	0.356
25–29	1.14 (0.83–1.57)	0.387	1.11 (0.67–1.85)	0.672
30–34	1.27 (0.93–1.73)	0.128	1.35 (0.78–2.35)	0.277
35–39	1.27 (0.90–1.78)	0.161	1.33 (0.76–2.32)	0.317
40–44	1.22 (0.80–1.85)	0.346	1.66 (0.88–3.13)	0.113
45–49	0.49 (0.24–0.99)	0.050	1.17 (0.52–2.66)	0.692
Maternal education				
No formal education	Ref		Ref	
Primary school	5.26 (4.18–6.61)	< 0.001	1.46 (1.13–1.90)	0.004
Secondary school	22.56 (16.49–30.85)	< 0.001	2.38 (1.65–3.42)	< 0.001
Higher	151.05 (52.45–434.99)	< 0.001	2.46 (0.75–7.98)	0.133
Husband education				
No formal education	Ref		Ref	
Primary school	3.00 (2.25–3.99)	< 0.001	1.25 (0.91–1.71)	0.164
Secondary school	9.36 (7.12–12.28)	< 0.001	1.43 (1.03–1.99)	0.033
Higher	60.62 (33.38–110.11)	< 0.001	2.13 (0.90–5.03)	0.084
Place of residence				
Urban	Ref		Ref	
Rural	0.12 (0.08–0.18)	< 0.001	0.50 (0.33–0.74)	0.001
Religion				
Catholic	Ref			
Protestant	0.70 (0.51–0.95)	0.025	0.72 (0.51–1.02)	0.066
Other Christians	0.78 (0.50–1.22)	0.290	0.55 (0.35–0.87)	0.011
Muslim	0.31 (0.21–0.44)	< 0.001	0.75 (0.53–1.07)	0.120
Animist	0.08 (0.04–0.14)	< 0.001	0.46 (0.20–1.02)	0.057
Others	0.27 (0.14–0.54)	< 0.001	0.51 (0.24–1.09)	0.085
Economic status				
Poorest	Ref		Ref	
Poorer	3.63 (2.77–4.74)	< 0.001	1.45 (1.04–2.02)	0.025
Middle	8.73 (6.35–12.02)	< 0.001	1.88 (1.27–2.78)	0.002
Richer	28.30 (17.52–45.71)	< 0.001	2.71 (1.63–4.48)	< 0.001
Richest	126.08 (69.60–228.37)	< 0.001	5.16 (2.58–10.30)	< 0.001
Media exposure				
No	Ref		Ref	
Yes	10.00 (8.09–12.36)	< 0.001	1.46 (1.11–1.91)	0.006
Region				
Adamawa	Ref		Ref	
Centre (Without Yaoundé)	4.66 (2.45–8.86)	< 0.001	1.51 (0.79–2.88)	0.201
Douala	45.03 (19.96–101.59)	< 0.001	2.08 (0.78–5.51)	0.140
East	1.50 (0.84–2.68)	0.161	0.59 (0.33–1.04)	0.072
Far-North	0.75 (0.44–1.26)	0.283	0.79 (0.46–1.37)	0.411
Littoral (Without Douala)	28.78 (14.79–56.02)	< 0.001	10.89 (4.57–25.95)	< 0.001
North	0.73 (0.42–1.25)	0.256	0.94 (0.53–1.68)	0.859
North-West	9.82 (2.67–36.14)	0.001	3.23 (0.93–11.16)	0.063
West	42.69 (17.77–102.56)	< 0.001	9.86 (4.41–22.05)	< 0.001
South	5.80 (3.21–10.48)	< 0.001	1.19 (0.64–2.24)	0.571
South-West	138.01 (17.79–1070.53)	< 0.001	4.74 (0.62–35.82)	0.131
Yaoundé	14.37 (4.54–45.43)	< 0.001	1.20 (0.43–3.34)	0.726
Skilled ANC				
No	Ref		Ref	
Yes	30.20 (21.56–42.31)	< 0.001	14.46 (10.01–20.89)	< 0.001
Decision making				
No decision making	Ref		Ref	
Moderate decision making	2.51 (1.91–3.29)	< 0.001	1.12 (0.80–1.57)	0.479
Higher decision making	4.04 (3.02–5.40)	< 0.001	1.88 (1.36–2.59)	< 0.001
Attitude towards wife beating				
Accept	Ref		Ref	
Refuse	1.49 (1.21–1.83)	< 0.001	1.32 (1.05–1.65)	0.014
Parity				
1–2	Ref		Ref	
3–4	0.74 (0.63–0.87)	< 0.001	0.67 (0.48–0.93)	0.019
5+	0.47 (0.39–0.56)	< 0.001	0.62 (0.41–0.93)	0.024

*ref* reference

Married women who had skilled ANC visit were 14.5 times (aOR = 14.46, 95% CI; 10.01–20.89) more likely to use skilled childbirth services than those who had no skilled ANC visit. Decision making power was also strongly associated with skilled birth attendance. Married women who had decision making power, either alone or together with their husbands in all of the three decision making parameters (their own health, to purchase large household expenses, to visit family or relatives) had higher odds (aOR = 1.88, 95% CI; 1.36–2.59) of reporting skilled birth attendance in comparison with those who had no decision-making power. Attitude towards wife beating was observed to be associated with skilled birth services utilization, as women who refused wife beating had higher odds (aOR = 1.32, 95% CI; 1.05–1.65) of reporting skilled childbirth services than those who justified wife beating. Married women who had three to four delivery history were less likely (aOR = 0.67, 95% CI; 0.48–0.93) to use skilled childbirth services compared to those who had one to two delivery history.

## Discussion

Many women in low-and middle-income countries are still dying from preventable and treatable complications that occur during and following pregnancy and delivery. Skilled attendance at birth by professionals, as well as timely detection and management of obstetric complications are the most effective ways of saving new born and maternal lives [1]. There is evidence that maternal morbidity and mortality can largely be reduced by skilled birth services utilization [32, 33]. In this study, we comprehensively examined the coverage and predictors of skilled birth attendance among married women who had begun childbearing in Cameroon. We found that most of the women (66.2%) used skilled assistance during their recent delivery. The high prevalence of skilled birth attendance in the current study is similar but comparatively higher than the prevalence in 2017 [34]. The probable reason for the rise in skilled birth attendance in Cameroon could be due to the country’s effort to reduce maternal mortality associated with non-use of maternal healthcare services through increased training of health professionals who provide maternal health services [35] and financial support for programs aimed at increasing access to these services [36]. The finding implies the importance of encouraging skilled births in maternal healthcare in Cameroon. To enhance skilled birth attendance, the government of Cameroon should put in place strategies aimed at increasing the number of trained health professionals who provide maternal healthcare services. Programs should also be developed to engage community leaders in efforts to improve maternal health. These leaders can influence community members’ beliefs and behavior by encouraging the use of skilled birth attendants.

We found an association between level of education and skilled birth attendance. Our findings are in line with the findings of previous studies who concluded that women with higher levels of education were more likely to utilize the services of skilled birth attendance during delivery [22, 24, 25]. This finding is not surprising as it has been found that in Cameroon, the proportion of pregnant women who consult a doctor at least once increases with the level of education [37]. Once, they use the services of a doctor during pregnancy, they are also more likely to use the services of a doctor or other health professionals during delivery. This finding could be attributed to the fact that educated women are more likely to receive care during pregnancy and delivery than uneducated mothers [38], and are also believed to be informed on possible signs of obstetric danger [39], which allow them to seek prompt medical advice [39]. Moreover, education may influence women’s healthcare-seeking behavior by promoting health awareness and self-efficacy [40]. Findings on the association between level of education and use of skilled childbirth services indicates the need for government to provide access to education in the country, especially for the girl-child. Apart from the mainstream education in the classroom, media channels such as radio and television can be used as sources of information on the importance of skilled birth attendance for pregnant women. The findings call for the need to examine the geographical disparities in use of skilled childbirth services using decomposition analysis.

Furthermore, we observed lower utilization of skilled delivery services among married women in rural areas compared to their counterparts in the urban areas. This finding is consistent with previous studies in Ethiopia [23] and Bangladesh [41]. The possible explanation for this phenomenon may be due to lack of transport, poor access and cost [42, 43], and distance to health facilities [43]. In fact, rural women are less likely to use skilled delivery services because health facilities are located far way [43–45], and are more often closer to urban areas [42, 45]. In Cameroon, there are disparities between administrative regions and districts, and inadequate distribution of workforce may be the main challenge in the implementation of primary healthcare [46]. In the 2014 census report, it was noted that about 147 districts out of 181 had less than 50% of the staff required [46]*,* and these districts are in the rural areas [46]. Findings on the disparities in use of skilled childbirth services in rural an urban area shows geographical gap that can be bridged by ensuring access to skilled childbirth services for rural dwellers. As part of policy interventions to address the rural–urban gap in access to skilled birth attendance, the government of Cameroon, with the support of UNICEF rolled out the UNICEF Cameroon Country Programme 2018–2020 which among other things seeks to ensure that health facilities are made available to rural dwellers and that skilled health professionals are deployed to rural areas to provide healthcare services [47].

We found an association between religion and that skilled birth services utilization. In sub-Saharan Africa, studies have shown that women who are traditionalists are less likely to utilize skilled birth services due to their high inclination to their traditional beliefs, norms and practices [48–50]. Prior evidence have shown that spiritual practices are exercised before and during delivery by women or religious leaders, where anointing oil, blessed water, blessed white handkerchief, blessed sand, Bible and Rosary are used for a safe delivery[51–53]. The religious differences in skilled childbirth services utilization shows the role religious norms and practices play in the use of maternal healthcare services. This is an indication that, governmental and non-governmental organizations need to intensify education on maternal healthcare services utilization, taking into consideration the norms, values and beliefs that women share. Notwithstanding, future studies using qualitative research approach should be done to provide an understanding the reasons for the religious disparities in the use of skilled childbirth services.

The findings from the study further revealed that wealthier women were more likely to use skilled birth services compared to the poor, as reported in Ghana [24] and Bangladesh [41]. Delivery cost may be a critical barrier for the uptake of skilled birth services in many low-and middle-income countries [40], and some countries in sub-Saharan Africa have exempt policy for skilled birth services [54]. However, indirect cost including transport cost, loss of wage or earning may sometimes be higher than the direct cost of the service [55], leading to low utilization [40]. To achieve this, the governmental of Cameroon, with support of UNICEF and other non-governmental organizations has ensured reduction in cost of maternal healthcare services through the health insurance policy [47].

High utilization of skilled birth services was found among women who had media exposure compared to those without any form of exposure (i.e., television, radio and newspaper) [41]. Mass media exposure has been shown to uphold awareness of societal issues, and further improve the understanding of culture and beliefs [56–58]. Specifically, these studies found that women who are exposed to mass media are more likely to utilize maternal healthcare services including skilled birth attendance compared to those who are not exposed to media. Another crucial role of the media is that, it influences societal attitude and political view, changing cultures, business activities and increase awareness on health issues including maternal and child health [59]. It has also been established that information originating from the mass media are understandable to all receivers [60], which can make promotion and education on maternal health issues relatively less difficult [60, 61].

We found an association between region and skilled birth services utilization Workforce and facility density, quality of care, and availability of essential commodities at facilities can be the reasons for the inequality [21]. In Cameroon, the economically wealthier regions which are Centre, Littoral and West concentrated 11, 777 health care workers out of 19,709 overall country’s health care workers. More specifically, these regions took about 59.75% of the country’s health worker to serve 42.14% of the country’s total population which are found in those regions [46]. Women who attended ANC by skilled health worker were found to utilize skilled birth services [62, 63], because they may receive adequate information about pregnancy and childbirth related complications and treatment. A previous study in Cameroon noted that non-attendance of skilled ANC has led to the death of many women [64], as a result of post-partum hemorrhage, complication of unsafe abortion, ectopic pregnancy and other obstetric related complications [64]. Findings on the disparities in use of skilled childbirth services in poorer regions compared to richer regions shows geographical gap that can be bridged by ensuring access to skilled childbirth services for most disadvantaged regions in the country. The findings call for the need to examine the geographical disparities in use of skilled childbirth services using decomposition analysis.

Other factors related to women empowerment were found in this study, and others [25, 41] to be associated with skilled birth delivery. For instance, women with decision making power were found to use skilled childbirth services than those without decision making power. According to the 2018 Cameron DHS report, only 47% of married women had decision making power about their own health, major household purchases and to visit families or relatives and 31% had no decision power on all of the three parameters [15]. Meanwhile, previous studies in Western Ethiopia [18], Kenya [65], Ghana [66] and Benin [22] showed that women autonomy/decision making power may have positive impact on institutional delivery [18, 66], as well as skilled childbirth services [22, 65]. Furthermore, women who “refused” wife beating were more likely to use skilled childbirth services than those who justified wife beating [67].

In Cameroon, approximately 30% of women justified wife beating by their husband [15]. Evidence suggests that women who are abused by their partners may suffer from physical damage and mental health problems including higher levels of depression, anxiety and phobias than non-abused women [68]. Nonetheless, women who consider such violence ‘justifiable’ have been shown to be controlled by their partners, even though violent means [69]. Prior studies found that skilled ANC and skilled childbirth services utilization were lower among women who experienced intimate partner violence [67]. It has also been established that women who consider violence as ‘unjustifiable’ were more likely to be aware of their sense of entitlement, self-esteem, status [70], which may reflect positively on their empowerment status [69, 70]. Findings on the role of decision-making and justification of wife beating on the use of skilled childbirth services support the need to empower women through education which can help them to be much involved in decision-making and change their perceptions towards negative socio-cultural norms. Doing this will be the benchmark for their use of maternal healthcare services, including skilled childbirth services.

Our analysis also highlighted the effect of parity on skilled childbirth services. Having more children was associated with low utilization of skilled childbirth services. This findings is consistent with other studies in Ethiopia [18] and Nigeria [71]. Prior reported that higher parity women may not use ANC as recommended because of their increased self-confidence from experience of previous pregnancy and childbirth, or due to time and resource constrains caused by a larger family [72, 73].

### Strengths and limitations

The major strength of this study was that use of a recent nationally representative DHS data from Cameroon. However, some limitations were also observed. First, the data was cross-sectional and therefore no causality can be inferred. Second, since information were self-reported, the chances of recall and reporting bias cannot be overlooked [74]. Third, other factors such as cultural factors, attitude of health care providers, and expectation and quality of care that could predict skilled birth services utilization were not included in the model due to data limitations.

## Conclusion

This study demonstrates that social, economic, regional, and cultural factors can act as barriers to skilled birth services utilization in Cameroon. Interventions that target women empowerment, ANC awareness and strengthening are needed, especially among rural poor, to reduce barriers to care seeking. Maternal healthcare services utilization interventions and policies in Cameroon need to focus on specific equity gaps that relate to socio-economic through maternal education and the economic empowerment of women. Such policies and interventions should also aim at reducing geographical barriers to access to maternal healthcare services, including skilled birth attendance. Due to the presence of inequities in the use of skilled attendance services, programs aimed at social protection and empowerment of economically disadvantaged women are necessary for the achievement of the post-2015 targets [75] and the Sustainable Development Goals (2).

## Data Availability

Data for this study were sourced from Demographic and Health surveys (DHS) and available here: http://dhsprogram.com/data/available-datasets.cfm.

## Abbreviations

CI: Confidence interval
DHS: Demographic Health Survey
MMR: Maternal mortality ratio
OR: Odds ratio
SDG: Sustainable Development Goal
SSA: Sub-Saharan Africa
